# Influence of distributive justice on organizational citizenship behaviors: The mediating role of gratitude

**DOI:** 10.3389/fpsyg.2022.974405

**Published:** 2022-09-30

**Authors:** R. Bala Subramanian, P. B. Srikanth, Munish Thakur

**Affiliations:** ^1^Rajagiri Business School, Rajagiri College of Social Sciences, Kochi, India; ^2^Xavier School of Management, Jamshedpur, India

**Keywords:** gratitude, distributive justice, organizational citizenship behavior (OCB), social exchange theory, positive emotion

## Abstract

Distributive justice is known to have important emotional and affective outcomes. The present study explores the role of distributive justice as an antecedent to feelings of gratitude toward the organization. Borrowing from social exchange theory, we investigate the mediating role of gratitude in the relationship between “perceived fairness in distributive justice” and “employees’ organization citizenship behaviors (OCB).” Time-lagged, multi-source data was collected from 185 employees and their supervisors employed in a large manufacturing organization based in East India. Two significant findings emerge. First, the results indicate that feelings of gratitude signal fair distribution of benefits such that the employees go beyond the call of the duty to invest in OCB. Second, engagement in such acts seems to nullify their social debts highlighted in the social exchange perspective. Thus, a strong moral emotion, gratitude is a powerful vehicle that drives employees to act in the organization’s interests because doing is desirable and rightful. Implications for theory and practice are discussed.

## Introduction

Over two decades of research has established that gratitude positively impacts personal wellbeing (Watkins et al., 2019), and social wellbeing (Tsang and Martin, 2019). Literature examining the positive outcomes of gratitude is well documented (Cameron et al., 2011; Spence et al., 2013). As a vital emotional resource, gratitude is defined as “a generalized tendency to recognize and respond with grateful emotion to the roles of other people’s behavior in the positive experiences and outcomes that one obtains” (McCullough et al., 2002, p. 112).

In order to understand and appreciate the definition, it is noteworthy to highlight two salient aspects of it. First, the manifestation of gratitude is unconstrained and unrestricted by the magnitude of the effort it takes to express their thanksgiving (Emmons et al., 2019; Tsang and Martin, 2019). Second, response to the benefits received is based upon the “experience” of the perceived benefits one receives. Put differently, expression of gratitude manifests in behavior that exceeds the call of duty, scope of responsibility, boundaries of one’s work domain, and the norms of reciprocity (Algoe et al., 2013; Bock et al., 2016; Di Fabio et al., 2017; Fehr et al., 2017).

Events are known to trigger not just cognitive evaluations but also emotional appraisals (Cropanzano et al., 2019). Individuals not only think but also feel about their experiences at the workplace. While the cognitive responses to events of perceived justice have been studied extensively (Colquitt and Zipay, 2015), understanding of the emotional response that stems from justice-related outcomes remains inchoate (Verma and Yu, 2019). Despite the widespread speculation that moral behavior evokes moral emotions of gratitude, we came across only two studies (Ford et al., 2018; Verma and Yu, 2019) that have examined the relationship between organizational justice and gratitude. Of these, Wang et al. (2018) examined the combined relationship of gratitude (positive emotion) and anger (negative emotion) on interactional justice. Studied the relationship of three justice types (procedural, distributive, and interactional justice) with work engagement mediated through positive emotions- pride and gratitude.

Because the judgment about justice being fair is not cast in stone, employees may be inclined to use their feelings as a proxy for attributing fairness to justice-related events. Emotions can help seal the gap between objective justice and perceived justice. Thus, responses such as “I don’t think that the outcomes are fair” are emotionally laden, reflecting how people feel about events; rather than mirroring their thoughts associated with such events (Colquitt and Zipay, 2015). Importantly, distributive justice, defined as the “fairness of the decision outcomes” (Colquitt et al., 2013), may hold the key to whether employees consider their organizations fair. Such feelings can evoke employee gratitude (Fehr et al., 2017; Kersten et al., 2021).

On one hand, perceptions of distributive justice signal worthiness to its recipients—whether (or not) the organization values their contribution. On the other, employees also assess whether the organization’s distribution decisions went to deserving employees. In an organization, employees experience and express gratitude for a multitude of reasons (Locklear et al., 2022). Such reasons may include the opportunity to develop job-relevant technical skills, promotion to a higher grade, spot awards, exposure to working with top management teams, with senior members in the organization, or getting nominated for a marquee leadership development program. Recent studies have shown that individuals express gratitude not only to other individuals but also to the organization they work for Fehr et al. (2017), Jimenez (2018), Chen et al. (2020). Individuals infer the accuracy of deserving decisions by evaluating whether the rightful employees were rewarded.

In sum, this paper aims to examine the instrumental role of moral emotion originating from perceived fairness associated with distributive justice. First, while previous studies speculate that moral acts (e.g., distributive justice) may evoke moral emotions (e.g., gratitude), such claims lack empirical support (McCullough et al., 2002; Verma and Yu, 2019). Second, our understanding of whether the source of gratitude determines the direction of employees’ discretionary behaviors remains unknown (Spence et al., 2013). A granular investigation of the perceptions of distributive justice and their association with gratitude can shed light while addressing the gaps.

We contribute to gratitude and justice literature in several ways. We consider the lack of attention paid toward perceptions of distributive (in) justice as an antecedent to gratitude as a gap, given that the justice perceptions may influence employees’ voluntary behaviors benefitting the organization. Drawing upon social exchange theory (Blau, 1987), we suggest that employees feel obligated to volunteer for organizational cause when they perceive reward distribution norms as fair. When employees perceive their organization as fair, they go beyond reciprocity norms to benefit the organization by working harder. Our views are consistent with the affective component of social exchange theory that posits that moral emotions stem from moral actions and behaviors (McCullough et al., 2001b; Malti et al., 2020; Septianto et al., 2020). The following sections present the theoretical support and develop study hypotheses.

## Influence of organizational justice on organization citizenship behaviors-o—a social exchange perspective

More than half a century of research on social exchange signals the role of positive emotions that play a role in influencing voluntary actions targeted toward the benefactor (DeCremer, 2007; Fortin et al., 2015; Wang et al., 2021). Studies show a positive relationship between organizational justice and organization citizenship behaviors (OCB) (Rahman and Karim, 2022). Meta-analytic report (Colquitt et al., 2013) endorses that distributive justice may significantly influence OCB directed toward the organization (OCB-O).^1^

Justice perceptions stemming from exchange dealings can evoke positive or negative emotions (Lawler and Thye, 1999; Wu et al., 2017). For instance, these perceptions determine affective-emotional states and corresponding responses that resonate with (in) justice (Moorman, 1991; Cropanzano and Mitchell, 2005).

Social exchange theory (Blau, 1987) posits that following the norms of reciprocity, individuals reciprocate positively or negatively for the benefits/harm they perceive. “Social exchange. refers to voluntary actions of individuals that are motivated by the returns they are expected to bring and typically do bring from others.” (Blau, 1987, p. 91). Existing research has established the instrumentality of social exchange to understand dyadic relationships for reciprocal or resource exchange behaviors. The norms of reciprocity indicate that beneficial actions by one party would lead to a similar response by another party in a bilateral relationship (Cropanzano and Mitchell, 2005). Recent studies have shown that the receivers feel obligated to respond by returning tangible and intangible favors. Such favors can be social (e.g., influence, status, and love) and economic (e.g., money, information, goods, and service) (Muthusamy and White, 2005).

In the present study context, we consider perceptions of distributive justice from where tangible outcomes originate (Cohen, 1987; Leclercq et al., 2020) to form a fertile ground for the employee to experience and express their gratitude toward the organization. This form of gratitude stems from the desire to preserve and enhance the person’s interest (e.g., supervisor) by paying his/her debts for the valued resources (outcomes) received.

Research suggests that when employees perceive their authority figures to be acting morally, such actions are likely to boost feelings of gratitude (Jiang and Qu, 2022) for two reasons. First, employees are likely to consider themselves fortunate to be part of an organization that emphasizes that its managers appear fair and transparent in allocating benefits (Cropanzano et al., 2019; Estreder et al., 2020). Second, being a beneficiary may boost employees’ self-esteem since they are recognized for their valued contributions (Haider et al., 2019). Third, even if some employees do not receive any benefits *via* the social exchange, they might feel gratitude because distributive justice establishes norms of inclusion and exclusion in a manner that those worthy of benefits are visible and known (Lawler and Thye, 1999). “Gratitude results from the attribution of positive events to others” (Lawler and Thye, 1999); individuals could feel grateful so long as they perceive benefits to be awarded to deserving others.

This implies that if the employee believes that the organization fairly allocates rewards, he/she is likely to expend efforts in ways that benefit the organization (Yaakobi and Weisberg, 2020). By doing so, a grateful employee can express his positive feelings toward the organization of which the authority figure is a prominent part. By engaging in OCB-O, the employee perceives that s/he has repaid the moral debts that s/he owed to the organization, fulfilling the norms of social exchange.

## Hypothesis development

### Impact of distributive justice on organization citizenship behaviors– O

Despite the emergence of myriad theoretical perspectives, scholars agree that social exchange entails a multitude of interactions that create obligations (Emerson, 1987; Cropanzano and Mitchell, 2005). More importantly, these interactions are embedded within social exchanges resulting in corresponding actions that resonate with actions from where the behaviors originate (Blau, 1987). Such corresponding transactions create high-quality social ties (Cropanzano and Mitchell, 2005). When employees perceive the actions of authority figures as fair, they feel obliged to reciprocate, fulfilling the social reciprocity norms. Fair actions may signal the legitimacy and trustworthiness of the organization because superiors act as custodians of policy and rules. In a related study, Aryee et al. (2002) reported that organizational trust mediated justice perceptions on organizational commitment, job satisfaction, and intentions to quit. We propose that fairness perceptions would encourage employees to give time beyond time to volunteer for OCB-O because doing so satisfies the needs of felt obligation (Eisenberger et al., 2001). As such, exchange relationships entail repayment within a given period. The repayment rules may stem from either local folklore or out of morality (Cropanzano and Mitchell, 2005). In either case, employees may act for the organization’s benefit because superiors appear transparent and trustworthy in their dealings. One way to repay moral debts is to act in ways that benefit others—including the organization.

*Hypothesis 1: Perceptions of distributive justice encourage employees to engage in OCB-O.*

### Indirect effects of distributive justice on organization citizenship behaviors -O *via* gratitude

Distributive justice is outcome-oriented and tangible (Cohen-Charash and Spector, 2001), wherein the beneficiary assesses the fairness of benefit/distribution. It is different from procedural justice, which is defined as “an individual’s perceived fairness of the rules applied to a decision-making process” (Colquitt, 2001, p. 386). Interactional justice is “an individual’s perceived fairness of interpersonal treatment during interactions, thus highlighting the notions of respect, politeness, honesty, and dignity one receives from others” (Luo, 2007, p. 647).

Given that distributive justice determines “who gets what” (Cohen, 1987), employees who perceive that they have received benefits that meet or exceed their expectations should feel gratitude (Sun et al., 2019). Since gratitude is a moral emotion, it creates a sense of moral obligations (Greenbaum et al., 2020) among people who socially “owe” to their benefactors. Such indebtedness provides the motivational drive for OCB-O (Fehr et al., 2020). By engaging in OCB-O, the employees’ actions would be consistent with the norms of reciprocity outlined in the moral exchange perspective (Bergeron and Thompson, 2020).

Any support the organization offers to rightful employees should trigger a moral obligation to reciprocate (Ritzenhöfer et al., 2019). If not reciprocated, the employee may perceive the social exchange as “unequal” and feel “indebted.” Hence, employees reciprocate the obligation through positive discretionary behaviors, such as OCB-O. This is in sync with the core premise of the social exchange theory (Jonkman, 2020). There is a positive relationship between justice perceptions and citizenship behavior (Organ, 1997; Masterson et al., 2000). The employee may “go the extra mile” to volunteer in organizational initiatives in reciprocation for the perceived “fair justice.”

We propose that distributive justice should positively relate to gratitude toward the organization due to the latter’s outcome focus. Managers as authority figures are vested with decision-making powers (both administrative and developmental), which they need to execute as a matter of their role prerogative. To function as effective managers, people with administrative responsibilities need to allocate rewards and incentives to their subordinates to motivate them to perform better. In other words, when others (both recipients and non-recipients of benefits) perceive those reward allocations are justified, they would intuitively presume that due procedures were followed to arrive at a benefit decision (Cropanzano et al., 2019). In contrast, if the outcomes are not seen as fair, the subjects would raise the alarm about the consistency of the procedures that were followed in arriving at the outcome decision (Colquitt et al., 2013). Feelings of gratitude originate from attribution judgments about fairness (Lawler and Thye, 1999). When employees perceive that the norms of distributive justice have not been flouted, they should feel grateful even if they are not direct recipients of the benefits since the criteria to be classified as a beneficiary is evident and transparent. These sanguine views are consistent with the affective component of moral exchange theory (Blau, 1987), which posits that moral emotions stem from moral actions and behaviors.

Justice-related events follow positive or negative emotions based on fairness perceptions (Colquitt and Zipay, 2015). Literature has considered distributive justice to be “cold” given the outcome-directedness (Jonkman, 2020; Leclercq et al., 2020). However, employees’ fairness perceptions are not only based on “cold” cognitive aspects but also on “hot emotion-laden” responses to events (Colquitt and Zipay, 2015). Employees not only think about the “unfairness” but also feel it is unfair. When benefits are awarded to deserving employees, it signals that authority figures in the organization have been fair and consistent in passing rewards to worthy employees whose contributions are valued by the organization (Cropanzano et al., 2019). Because moral behavior is known to be associated with moral emotions (Colquitt and Zipay, 2015), when employees consider that authority figures of the organization have moral ways, they should feel grateful. Recipients of benefits should naturally feel grateful for what they receive; the non-recipients should feel equally grateful that the benefits went to deserving employees and that the organization is fair and transparent in allocating benefits.

Further, research illustrates that when people report moral feelings of gratitude, they feel obliged to repay what they owe, following the norms of reciprocity that underlie rules for social exchange (Spence et al., 2013; Cropanzano et al., 2019; Wang and Koerber, 2020). Since supervisors are bona fide representatives of the organization, employees would want to reciprocate positively by working harder and going beyond duty to repay the debts they presumably owe. Stated formally, we hypothesize:

*Hypothesis 2: Feelings of gratitude toward the organization will mediate the relationship between employee perception of distributive justice and their subsequent engagement in OCB-O*

## Materials and methods

### Sample and procedure

The study was conducted in a large manufacturing organization based in East India. The Human Resource (H.R.) department was keen to know the employees who are most likely to volunteer for community welfare activities as they had undertaken a community development project for a cluster of villages surrounding the manufacturing facility. We recruited the participants by asking them to volunteer for a study to capture their views of organizational practice. One of the authors was associated with this organization at the time of data collection. An online survey collected the data from employees and their superiors, across two periods. The H.R. Deptt provided the email ids for the employees and their supervisors. We sent an email with a covering note explaining the study’s purpose and assured the response’s confidentiality. The H.R. department identified the employees and supervisors. Both the employees and supervisors completed the survey during work hours. Of the 434 employees, 203 employees submitted the survey. The elimination of missing values gave us 195 usable data points (a 45 percent response rate). We then emailed 160 supervisors with whom these 195 employees had a direct reporting relationship. We received completed surveys from 150 supervisors (94 percent response rate). Thus, our final sample comprised 185 employees with corresponding responses from their supervisors.

The employees’ average age was 32 years (s.d. = 4.5). The employees’ average tenure in the organization (OG) was 2.43 (s.d = 1.20) (Table 1). There were 33% female respondents, and the rest were males.

**TABLE 1:** Estimated sample statistics for the latent variables.

Variable	Mean	*SD*	1	2	3	4	5	6
1. Gender	1.33	0.47						
2. Age	32.00	4.50						
3. Tenure^+^	2.43	1.20						
4. DJ (T1)	3.43	0.72	–	–	–	1.000		
5. OCB-O (T2)	3.85	0.59	–	–	–	0.32**	1.000	
6. OG (T1)	3.78	0.71	–	–	–	0.55**	0.38**	1.000

*N*, 185. T1, Time 1; T2, Time 2; **p < 0.01; ^+^Tenure was an ordinal variable.

### Measures

#### Gratitude to the organization

Studies have contextualized gratitude toward the organization, coach, and sports team (Chen and Kee, 2008; Akgün et al., 2016; Chen and Chang, 2017). For the present study, we measured employees’ gratitude toward their organization. At time 1, employees completed a 10-item measure of gratitude toward the organization. The scale was a modified version of The Gratitude Questionnaire (GQ-6) of McCullough et al. (2001a) and the Gratitude, Adjective (GAC) of McCullough et al. (2002). The sample item includes “When I think of my organization, I feel a sense of gratitude.” Employees responded on a five-point Likert scale (“1”–never, “5”–always). After checking for reliability (α = 0.94) and validity, the item scores were added to form overall measures of gratitude to the organization.

#### Distributive justice

At time 1, employees completed a five-item measure of Niehoff and Moorman (1993) distributive justice scale. The sample item includes “I think that my level of pay is fair.” Employees responded on a five-point Likert scale (“1”–strongly disagree, “5”–strongly agree). After checking for reliability (α = 0.77) and validity, the item scores were added to form overall measures of distributive justice.

#### Organizational citizenship behavior directed toward the organization

At time 2, supervisors completed a five-item measure of Lee and Allen (2002) organizational citizenship behavior directed toward the organization about their subordinates. The sample item includes “This employee keeps up with developments in the organization.” Supervisors responded on a five-point Likert scale (“1”–never, “5”–always). After checking for reliability (α = 0.80) and validity, the item scores were added to form overall measures of organizational citizenship behavior directed toward the organization.

Higher scores indicate a higher value for all the constructs.

**FIGURE 1:**
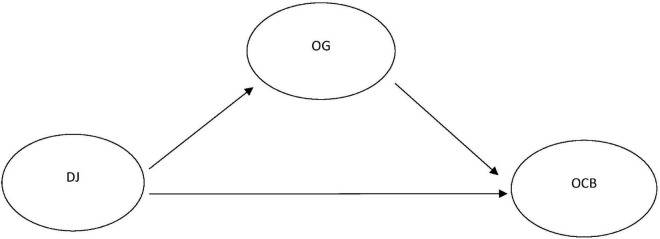
Proposed model.

The proposed model is presented here (Figure 1).

**FIGURE 2:**
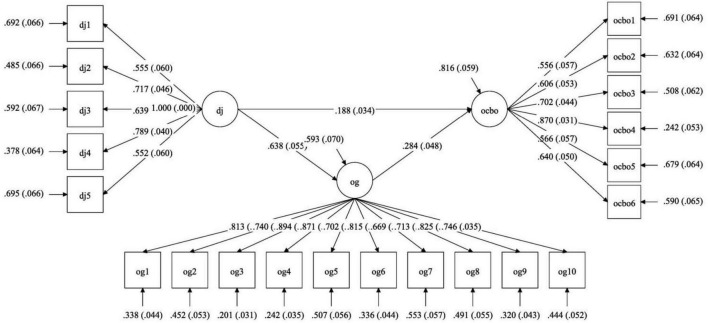
Summary of SEM result. Dj, distributive justice; Og, Gratitude toward organization; OCB-O, organizational citizenship behavior toward organization.

## Results

First, we conducted factor analysis using Mplus 8.3 (Muthén and Muthén, 2017). to verify that the items load appropriately to the desired constructs. We compared hypothesized three factors solution with two factors solution and a single-factor solution. Model fit was assessed through the root-mean-square error of approximation (RMSEA), the Tucker–Lewis Index (TLI), and the comparative fit index (CFI) as per the recommendations of the researchers (Hu and Bentler, 1999; Steiger, 2007; Hayes, 2012). The Table 2 shows that the three-factor solution (χ^2^ = 218, DoF = 102 and *p*-value < 0.01) is superior to two factors (χ^2^ = 330, DoF = 118 and *p*-value < 0.01) and single factor solution (χ^2^ = 554, DoF = 135 and *p*-value < 0.01).

**TABLE 2:** Factor solution.

Model	N of parameters	Chi square	DoF	*P*-value
One factor	54	554	135	0
Two factors	71	330	118	0
Proposed three factors	87	218	102	0
**Models comparisons**				
One factor vs. two factors	–	221	17	0
Two factors vs. three factors	–	114	16	0

After accepting the three factors solution, the correlation table and descriptive statistics were calculated (Table 1).

Table 3 presents the reliability and validity. The Cronbach’s alpha value (Table 3) for all 3 constructs were above 0.7 (DJ = 0.77, OCB-O = 0.80, and OG = 0.94). The average values extracted (AVE) for the distributive justice (DJ), OCB, and OG were 0.46, 0.51, and 0.61, respectively. Since AVE was less than 0.5 for DJ, the composite reliability was checked to see if DJ fulfills the criterion of construct validity. The composite reliability for DJ, OCB, and OG were 0.77, 0.80, and 0.94, respectively. Though the AVE for DJ was slightly lower than 0.5, its C.R. was higher than 0.7, suggesting that DJ can be considered a valid construct.

**TABLE 3:** Reliability and validity.

Indicator	DJ	OCB	OG
Cronbach’s alpha	0.77	0.80	0.94
Composite reliability	0.77	0.80	0.94
Average variance extracted	0.46	0.51	0.61

After establishing the reliability and validity, regression analysis was conducted to validate the hypotheses. Since demographic variables can influence the perception of justice, gratitude and OCB, control variables were added to the analyses. Age, tenure, and gender were used as control variables. Table 4 presents the values of regression analysis.

**TABLE 4:** Regression analysis.

	Dependent variable: OCB									Dependent variable: OG					
		
	Model 1			Model 2			Model 3			Model 4			Model 5		
					
Independent variables	Unstd B	S.E.	95.0% CI [B]	Unstd B	S.E.	95.0% CI [B]	Unstd B	S.E.	95.0% CI [B]	Unstd B	S.E.	95.0% CI [B]	Unstd B	S.E.	95.0% CI [B]
***Intercept***	3.91	0.05	[38.0–4.0]	2.57***	0.24	[2.09–3.0]	3.03***	0.21	[2.6–3.4]	3.82	0.07	[3.6–3.9]	1.98	0.22	[1.5–2.4]
***Age***	0	0	[0.00–0.00]	0	0	[0.00–0.00]	0	0	[0.00–0.00]	0	0	[0.00–0.00]	0	0	[0.00–0.00]
***Tenure***	0.01	0	[0.00–0.00]	0	0	[0.00–0.00]	0	0	[0.00–0.00]	0	0	[0.00–0.00]	0.01	0	[0.00–0.00]
***Gender***	0	0	[0.00–0.00]	0	0	[0.00–0.001]	-0.001	0	[0.00–0.00]	0	0	[0.00–0.00]	0	0	[0.00–0.00]
***DJ***				0.26***	0.05	[0.14–0.37]	0.1	0.058	[-0.00–0.22]				0.53***	0.062	[0.41–0.65]
***OG***							0.23***	0.069	[0.094–0.36]						
***R Sqr (Adj.)***	0.07			0.1			0.15			0.001			0.29		

***p < 0.001.

We used multiple hierarchical regression for analyzing hypotheses. In Model 1, OCB as a dependent variable was regressed with control variables, but none of them turned out to be significant (*b* = 0.00, s.e = 0.0). In Model 2, DJ was added as an independent variable to see the incremental effects of it on the dependent variable. The results (*b* = 0.26, s.e = 0.58, *p*-value < 0.001) shows that the relationship between DJ and OCB was significant with a positive slope, which means with the increase of DJ, OCB-O also increases. In Model 3, gratitude was added as an additional independent variable. Addition of OG led to change in strength of DJ-OCB relationship (*b* = 0.1, s.e = 0.058, *p* = 0.1). OG was also positively and significantly related to OCB (*b* = 0.23, s.e = 0.069, *p* < 0.001). In Model 4, the dependent variable was changed to OG from OCB, and control variables were considered independent variables. In Model 5 DJ, was added as an independent variable of interest in addition to control variables. The results prove that DJ is positively and significantly related to OG (*b* = 0.53, s.e = 0.062, *p* < 0.001).

In the hypothesis, we predicted a positive relationship between distributive justice, and OCB-O toward the organization, mediated by gratitude. To examine the mediating role of gratitude, the indirect effects of DJ on OCB was calculated using process macros suggested by Preacher and Hayes. We used process macros with Mplus to obtain indirect effects. Table 5 reports the results of direct, indirect and mediation.

**TABLE 5:** Indirect effects analysis.

Dependent variable: OCB					

Direct, indirect and total effects					

Independent variable: DJ		Mediator: Gratitude			

	Coefficient	*P*-value	S.E.	*P*-value	[CI-95%]
Indirect effects (H2)	*Unstandardized coefficient*	0.12	0.044	0.012	[0.012–0.21]
Indirect effects (H2)	*Standardized coefficient*	0.16	0.058	0.012	[0.054–0.28]
Total effects	*Unstandardized coefficient*	0.22	0.054	0.001	[0.11–0.32]
Direct effects (H1)	*Unstandardized coefficient*	0.092	0.063	0.14	[-0.03–0.21]

The mediation analysis suggest that the total effect is significant (*b* = 0.22, s.e = 0.054, *p* = 0.001) and the indirect effect (*b* = 0.16, s.e = 0.058, *p* = 0.012) is significant as well but direct effect was found to be insignificant (*b* = 0.092, s.e = 0.063, *p* = 0.14). These results indicate that gratitude fully mediates the relationship between distributive justice and OCB-O.

The SEM model in which distributive Justice and OCB–O are associated through gratitude showed a good fit. The results suggested a good fit on most indices (CFI = 0.920, TLI = 0.904, SRMR = 0.06, RMSEA = 0.072, Chi-Square value = 365.720). While a value of more than 0.95 is ideal for CFI and TLI, (Hu and Bentler, 1999), a value above 0.90 is also considered acceptable for CFI (Hooper et al., 2008) and TLI (Forza and Filippini, 1998).

The general rule for a good fit of RMSEA is a value closer to 0.06 (Hu and Bentler, 1999), an astringent range of 0.06–0.08 is considered acceptable (Steiger, 2007; Hayes, 2012).

Since the total effects of DJ on OCB-O were significant, it lent support to our first hypothesis. Similarly, significant indirect effect (*b* = 0.346, s.e = 0.138, *p* = 0.012) lends support to the critical hypothesis (H2), suggesting that OG mediates the relationship between DJ and OCB-O.

## Discussion

The study’s purpose was to investigate the association between moral acts (e.g., distributive justice) and moral emotions (e.g., gratitude). Based on the moral affect aspect of the social exchange theory, perceived fairness in distributive justice evokes a higher level of gratitude toward the organization among the employees. Distributive justice is more relevant because the employees are more concerned about the consistency in allocating benefits. Our results suggest that the perceptions of distributive justice induced moral obligation to pay back to the organization, among employees. The employees go beyond the transactional norms of reciprocity.

Consistent with our main hypothesis, gratitude mediated the relationship between employees’ perception of distributive justice and OCBO. Feelings of gratitude prompt the beneficiaries to repay their social debts by indulging in actions beyond the job description. The results are in sync with the previous studies that examined the moral emotion of gratitude and organizational justice (Verma and Yu, 2019; Ford et al., 2018) and between gratitude and OCBO (Spence and Brown, 2012; Spence et al., 2013). It is interesting to note that gratitude originating from distributive justice not only signals consistency in the allocation of benefits by the organization but also drives employees who may be beneficiaries to go beyond the call of duty to demonstrate OCBO.

### Theoretical implications

The article contributes to understanding employee gratitude resulting from the work environment. First, we examined distributive justice as an antecedent to employees’ feelings of gratitude toward their organization. When employees develop a perception of fairness in an organization, they tend to feel grateful to the organization (not only to the supervisor). Our results suggest that a fairness perception can also decide the directionality of gratitude feelings. It is equivalent to “counting the blessings” in the workplace (Layous, 2019).

Second, studies of gratitude in the organization have always been conceived as independent variables (Greenbaum et al., 2020), and the potential source of antecedents for gratitude in the context of the organization has been less explored (Ford et al., 2018; Guan and Jepsen, 2020). Our results extend organizational justice literature by suggesting that presence of distributive justice may induce feelings of gratitude among employees. Because distributive justice is outcome-driven, the implications are far-reaching. Thus, as a moral emotion, gratitude is the outcome of the “fair distribution of reward” in the workplace.

Third, employees tend to act positively toward the organization when they feel grateful. In line with the “moral reinforcement function” of the moral affect theory of gratitude (McCullough et al., 2002), grateful feelings reinforce a prosocial behavior toward the helper. Hence the employees volunteer in OCBO. By acting in instrumental ways to “give-back” to the organization *via* OCBO, gratitude is a powerful emotion through which employees display reciprocity behaviors outlined by the affect aspect of social exchange theory. Put differently, feelings of gratitude among employees may benefit the organization beyond what one intuitively expects through the transactional exchange.

### Managerial implications

Since employee engagement is critical for an organization, our findings suggest that gratitude in employees may not only signal consistency of moral acts by managers but also motivate them to go beyond the call of duty. Research suggests that grateful employees engage in voluntary discretionary effort and act as custodians for the organization. They feel connected to the organization, are more engaged in producing high-quality work, and contribute actively to OCB-O (Turner, 2020).

A larger managerial implication is on highlighting distributive justice in all forms of organizational rewards and benefits. One way to this end is to communicate the criteria used to decide the allocation of rewards. More importantly, managers need to explain the exclusion criteria to vindicate who was not entitled to the benefits and for what reasons. Understandably, the beneficiaries may feel grateful for what they have received. However, for those who couldn’t be included in the beneficiary list, it is quite natural for them to experience negative emotions. Managers need to explain the reasons in objective ways why they couldn’t be considered. A tangible measure of success will empower employees with the knowledge of what needs to be done to succeed. The non-recipients of the beneficiaries need to be convinced that they did not deserve benefits. Employees may feel grateful to the organization for providing useful feedback and explanation for offering a detailed action plan for further improvement. In doing so, the non-recipients of benefit may consider distributive justice fair and transparent, one that exudes the deservingness of benefits among various employees.

Distributive justice as a source of gratitude emotion may have far-reaching effects across various H.R. practices, not limited to compensation and benefits. For instance, those chosen for a specific leadership role as part of the succession plan may feel grateful, while others who were under consideration but not selected may feel left out. Management needs to offer a valid explanation to employees who were equally talented but not selected for leadership roles (say) as part of the succession plan. For example, they can be given alternative positions such as subject matter experts or leading a greenfield project. In doing so, organization authorities can ensure that talented employees don’t feel left out. Employees should value the effort taken by their reporting managers in making a strong business case for them, even if such efforts do not yield the expected results (McCullough et al., 2001a). Similarly, employees who aren’t selected for the leadership development program may be offered a real-time project to feel that their contributions are equally valued. Put differently, employees can be “nudged” to count their workplace blessings.

Managing employee perceptions is critical in organizations. Employees may make “fairness evaluations” almost daily, given that distributive justice is outcome-driven. The findings suggest that employees feel grateful when they perceive distribution as fair. The results imply that organizations must make distribution fair, transparent, and inclusive. Fair distributional procedures will help form the right expectation of fairness. One way to promote fairness is to involve employees in forming fairness policies. When employees are involved in the process and organizations consider their input in designing the performance management system, there will be fewer chances of employees’ grievances, leading to higher fairness perception and more gratitude.

By ensuring transparency and consistency in evaluations that affect outcomes, managers can ensure that employees of all types (not just beneficiaries) feel gratitude toward the organization and volunteer for different initiatives. Feelings of gratitude may supersede even the tangible rewards that organizations allocate. Such feelings among employees may create a more engaged workforce interested in acting in the organization’s interests.

### Limitations

The study is not without limitations. The data were collected from different stakeholders but not at the same time. Gratitude to the organization and perceived justice were measured at time T-1 from the employees. The supervisor at the time T-2 rated the OCB-O. But how much change in the perception of distributive justice or gratitude can’t be attributed to change in OCB-O. This is one limitation.

Second, this is limited to one organization that constraints the generalizability of the data findings. Third, we didn’t measure the effect of culture. Results have shown that gratitude manifests differently amongst cultures (Appadurai, 1985; Oishi et al., 2019) such as Korea, Japan, and India. Another limitation is that we did not measure the effect of a culture that may have contributed to the feeling of gratitude.

Third, all four aspects of organizational justice (Procedural justice, interactional justice, and informational justice) have an impact on employee gratitude and other organizational outcomes (Colquitt, 2001) because they are all interrelated in the employee’s cognition. Hence, the change in gratitude or OCB-O can’t be attributed solely to distributive justice. The other justice dimensions also might have interacted with distributive justice and thereby influenced the gratitude and or OCB-O. This is another limitation.

### Directions for future research

Future studies can look into the impact of the whole array of justice perceptions on gratitude, and OCB-O. As mentioned above, justice perceptions are interrelated. Studying them together will further expand our knowledge of their influence on gratitude.

Gratitude also influences team processes and outcomes (Pillay et al., 2020). We suspect justice perceptions may influence collective gratitude and their corresponding team outcomes. We consider this a logical extension of the present study. While our respondents included both the beneficiary and non-beneficiary, it will be useful for future research to examine if employees consider distributive justice fair, even if they don’t receive the benefit for a longer period.

Since distributive justice is outcome-driven, it may be useful to know how it affects daily gratitude and if employees volunteer for OCB-O daily. Subsequent studies may measure the variables as a daily diary study. We used a gratitude scale anchored around organization Scholars have talked about maintaining a gratitude diary (Leong et al., 2020). By collecting various behaviors from the gratitude diary of all employees, scholars can contextualize the measure of gratitude at a department or function level to capture nuances a generic gratitude scale may miss. We leave this thought for further studies.

## Conclusion

Distributive justice perceptions can anchor feelings of gratitude in ways that encourage employees to engage in voluntary actions beneficial to the organization. In doing so, the focal employee extends his/her moral emotions to a broader family—ultimately, the organization, whom the decision makers represent.

## Data availability statement

The raw data supporting the conclusions of this article will be made available by the authors, without undue reservation.

## Ethics statement

Ethical review and approval was not required for the study on human participants in accordance with the local legislation and institutional requirements. The patients/participants provided their written informed consent to participate in this study.

## Author contributions

All authors listed have made a substantial, direct, and intellectual contribution to the work, and approved it for publication.

## Footnotes

1 It is important to mention the similarities and differences between OCB and extra-role behaviors. Both OCB and extra-role behavior are similar as both are not expected or formally defined in the job description (Lee and Allen, 2002) and neither are they rewarded (Vandyne et al., 1995). However, the fundamental difference between the two is that while OCB is affiliative and promotive (Bergeron and Thompson, 2020), extra-role behaviors can be both promotive and prohibitive. Stated differently, OCB-O is always beneficial to the organization (Tremblay et al., 2022) but extra-role behavior (“challenging” dimension) may put the organization at risk (Organ, 1997).
